# Successful retreatment with grazoprevir and elbasvir for patients infected with hepatitis C virus genotype 1b, who discontinued prior treatment with NS5A inhibitor-including regimens due to adverse events

**DOI:** 10.18632/oncotarget.24620

**Published:** 2018-03-23

**Authors:** Tatsuo Kanda, Shin Yasui, Masato Nakamura, Shingo Nakamoto, Koji Takahashi, Shuang Wu, Reina Sasaki, Yuki Haga, Sadahisa Ogasawara, Tomoko Saito, Kazufumi Kobayashi, Soichiro Kiyono, Yoshihiko Ooka, Eiichiro Suzuki, Tetsuhiro Chiba, Hitoshi Maruyama, Mitsuhiko Moriyama, Naoya Kato

**Affiliations:** ^1^ Division of Gastroenterology and Hepatology, Department of Internal Medicine, Nihon University School of Medicine, Itabashi-ku, Tokyo 173-8610, Japan; ^2^ Department of Gastroenterology, Chiba University, Graduate School of Medicine, Chuo-ku, Chiba 260-8670, Japan; ^3^ Department of Molecular Virology, Chiba University, Graduate School of Medicine, Chuo-ku, Chiba 260-8670, Japan

**Keywords:** hepatitis C virus, direct-acting antiviral failure, ombitasvir, ledipasvir, resistance-associated substitutions

## Abstract

**Background:**

Sustained virologic response (SVR) by interferon and interferon-free treatment can results in the reduction of advanced liver fibrosis and the occurrence of hepatocellular carcinoma in patients infected with hepatitis C virus (HCV). Recent interferon-free treatment for HCV shortens the duration of treatment and leads to higher SVR rates, without any serious adverse events. However, it is important to retreat patients who have had treatment-failure with HCV non-structural protein 5A (NS5A) inhibitor-including regimens. Combination of sofosbuvir and ledipasvir only leads to approximately 100% SVR rates in HCV genotype (GT1b), NS5A inhibitor-naïve patients in Japan. This combination is not an indication for severe renal disease or heart disease, and these patients should be treated or retreated with a different regimen.

**Case summary:**

Retreatment with HCV non-structural protein 3/4A inhibitor, grazoprevir, and HCV NS5A inhibitor, elbasvir, successfully eradicated HCV RNA in three patients with HCV genotype 1b infection who discontinued prior interferon-free treatments including HCV NS5A inhibitors due to adverse events within 2 weeks.

**Conclusion:**

Retreatment with the 12-week combination regimen of grazoprevir and elbasvir is effective for HCV GT1b patients who discontinue the HCV NS5A inhibitor-including regimens within 2 weeks. The treatment response may be related to the short duration of initial treatment, which did not produce treatment-emergent RASs.

## INTRODUCTION

Infection of hepatitis C virus (HCV) causes acute and chronic hepatitis, cirrhosis and hepatocellular carcinoma (HCC) [1]. Eradication of HCV could prevent the progression of liver diseases, and the elimination of HCV is important to reduce the occurrence of HCC [1–3]. In Japan, HCV genotype 1b (GT1b) is predominant [3]. Interferon-free treatment can eradicate HCV from almost all patients with chronic HCV GT1 infection [4]. Other than patients with cirrhosis previously treated with peginterferon plus ribavirin, decompensated cirrhosis and/or GT3 infection represent the “difficult-to-treat” patients [2, 4].

In Japan, 12-week combination regimens of the HCV non-structural protein 5A (NS5A) inhibitor ledipasvir (90 mg daily) and the HCV non-structural protein 5B (NS5B) inhibitor sofosbuvir (400 mg daily) can lead to higher sustained virologic response (SVR) rates at 95-100% in both direct-acting antiviral agent (DAA)-naïve patients and previous HCV non-structural protein 3/4A (NS3/4A) protease inhibitor-users infected with HCV GT1 [5, 6]. However, retreatment with this regimen for HCV GT1-infected patients who failed to respond to the combination of the HCV NS3/4A inhibitor asunaprevir and the HCV NS5A inhibitor daclatasvir could result in only 60-70% SVR rates in Japan [7, 8].

The second-generation macrocyclic HCV NS3/4A protease inhibitor grazoprevir (MK-5172) demonstrated subnanomolar activity against various HCV GTs NS3/4A proteases and variants resistant to first-generation HCV NS3/4A inhibitors *in vitro* protease enzymatic assays [9]. In HCV replicon assays, grazoprevir exerted high selective pressure. Elbasvir (MK-8742), a tetracyclic indole-based HCV NS5A inhibitor has a potent activity against HCV pan-GTs [10].

In a 12-week combination regimen of grazoprevir and elbasvir for treatment-naïve cirrhotic and noncirrhotic patients with chronic HCV GT1, 4 or 6 infection, 95% SVR12 rates (299/316) were achieved (92% with GT1a (144/157); 99% with GT1b (129/131); 100% with GT4 (18/18); and 97% with GT6 (68/70)) [11]. A Japanese study also demonstrated that SVR12 rates were 96.5% and 97.1% after a 12-week combination regimen of grazoprevir and elbasvir in noncirrhotic and cirrhotic NS5A inhibitor-naïve HCV GT1-patients, respectively [12].

The 12-week combination regimen of grazoprevir and elbasvir had a low rate of adverse events and 99% SVR rates (115/116) in patients infected with HCV GT1 and had stage 4-5 chronic kidney disease [13]. The 12-week combination regimen of grazoprevir and elbasvir plus ribavirin achieved 96.2% SVR rates (76/79) in HCV GT1-patients after failure of triple therapy containing an earlier-generation protease inhibitor [14]. The combination of grazoprevir and elbasvir, with or without ribavirin is safe and effective for patients with HCV GT1 or GT4 infections, although in patients with HCV GT1a, resistance-associated substitutions (RASs) before treatment can affect the SVR rates [15].

We recently reported that retreatment with ledipasvir and sofosbuvir is effective for HCV GT1b patients who discontinue the combination of daclatasvir and asunaprevir within 4 weeks [16]. In the present case series, we reported that the 12-week retreatment with grazoprevir and elbasvir is effective for HCV GT1b patients who discontinued the NS5A inhibitor-including regimens within 2 weeks.

## CASE 1

A 67-year-old man was diagnosed with an HCV GT1b infection 11 years ago and underwent tonsillectomy in his twenties. He did not receive blood transfusions or have tattoos but had experienced drug abuse at age 23. He was a social drinker with no family history of HCV infection. He had a medical history of apical hypertrophic cardiomyopathy, right ventricular hypertrophy, diabetes mellitus and chronic pancreatitis. He used insulin (24 units daily) and pregabalin (100 mg daily) for the control of blood sugar and pain, respectively. He also took a dose of tramadol hydrochloride and acetaminophen tablets as medicine to be taken only once for his pain.

**Figure 1:**
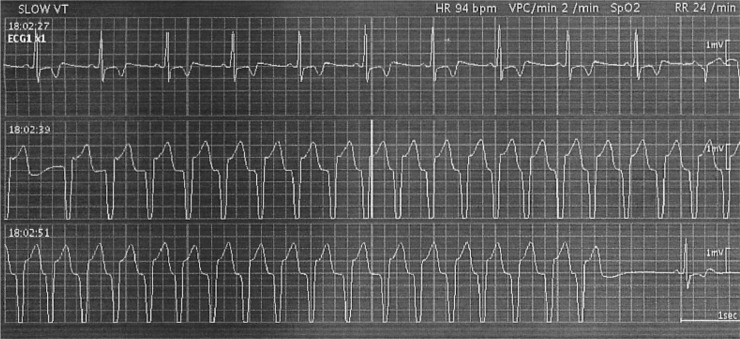
Ventricular tachycardia (VT) ran 2 days after the commencement of treatment with ledipasvir and sofosbuvir in Case 1 Stopping the treatment of ledipasvir and sofosbuvir, β-blocker was transiently used. This patient is now healthy without any prescribed drugs for cardiovascular diseases.

**Figure 2:**
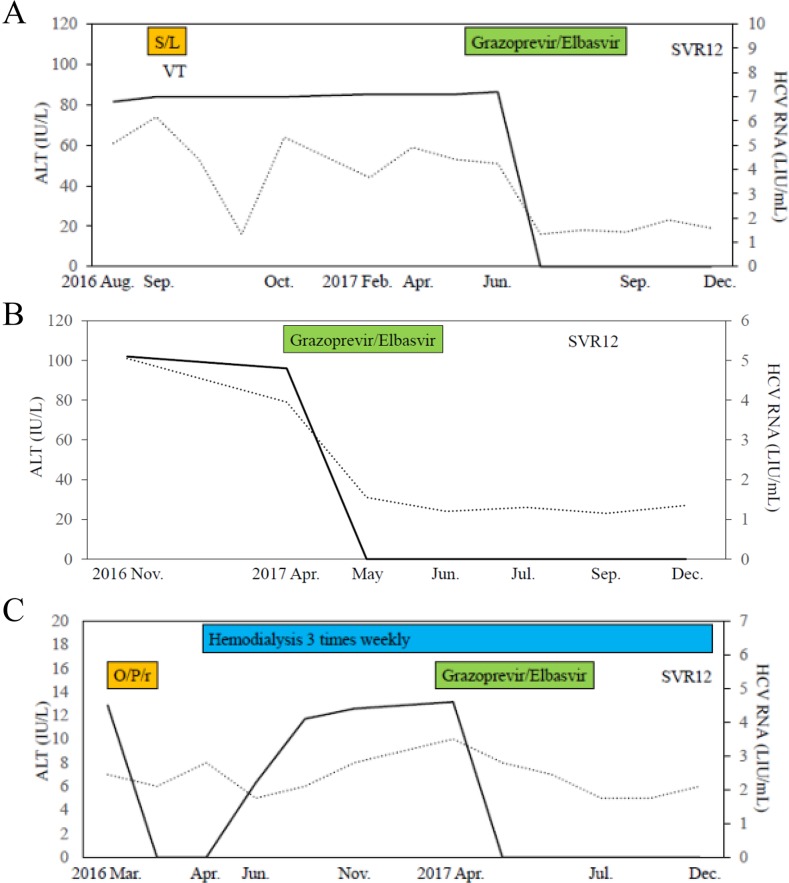
Clinical courses of 3 cases in the present study **(A)** Case 1, **(B)** Case 2, **(C)** Case 3. SVR12, sustained virologic response at week 12 after stopping treatment; VT, ventricular tachycardia; S/L, sofosbuvir/ledipasvir; O/P/r, ombitasvir/paritaprevir/ritonavir. Black lines and dot lines indicate HCV RNA and ALT levels, respectively.

He was an interferon-treatment naïve patient and was treated with ledipasvir (90 mg daily) and sofosbuvir (400 mg daily) in 2016 [6]. However, this treatment was discontinued after 3 days because of ventricular tachycardia (Figures 1 and 2A), and he required temporal β-blocker treatment. The laboratory data at the start of retreatment are shown in Table 1. The patient had no signs of cirrhosis. HCV NS5A-L31 and -Y93 sequencing using a real-time polymerase chain reaction (PCR) system and a cycling probe assay [17] revealed that he had Y93H at 33% as a HCV NS5A RAS. He willingly began retreatment with grazoprevir (100 mg daily) and elbasvir (50 mg daily) at 9 months after the initial DAA treatment. He received full doses of both grazoprevir and elbasvir for 12 weeks, and no adverse events were observed. The estimated glomerular filtration rate (eGFR) did not change during the therapy. Rapid virologic response (RVR) was achieved, with serum HCV RNA negativity at week 4 after commencing treatment. Finally, he achieved SVR at 12 weeks after the end of treatment (SVR12) (Figure 2A).

**Table 1:** Patients’ characteristics before the retreatment with grazoprevir plus elbasvir in the present study

Case	No. 1	No. 2	No. 3
Age (years)	67	49	76
Gender	Male	Male	Male
Previous interferon treatment	No	No	No
Prior interferon-free treatment	Ledipasvir/Sofosbuvir	Ledipasvir/Sofosbuvir	Paritaprevir/Ritonavir/Ombitasvir
Duration of prior treatment (days)	3	14	12
Adverse events in prior treatment	Ventricular tachycardia	HyperCPKemia	Renal dysfunction
Body length (cm)	169	173	165
Body weight (kg)	68	80	61.5
Body mass index (kg/m^2^)	23.8	26.7	22.6
White blood cell count (/μL)	4900	4400	7200
Red blood cell count (10^4^/μL)	471	447	393
Hemoglobin (g/dL)	14.9	14	12.2
Platelet counts (10^3^/μL)	198	56	85
Prothrombin time (%)	100	71	108
Total bilirubin (mg/dL)	0.5	2	0.5
Aspartate aminotransferase (IU/L)	40	63	17
Alanine aminotransferase (IU/L)	53	79	10
Lactate dehydrogenase (IU/L)	224	336	173
Alkaline phosphatase (IU/L)	228	308	387
γ-glutamyl transpeptidase (IU/L)	30	26	10
Total protein (g/dL)	7.6	7.2	7.5
Albumin (g/dL)	4.2	3	4.2
Blood urea nitrogen (mg/dL)	14	11	35
Creatinine (mg/dL)	1.04	0.69	7.64
Estimated glomerular filtration rates (ml/min/1.73 m^2^)	55.6	95.3	6.1
Blood sugar (mg/dL)	171	92	95
α-fetoprotein (ng/mL)	8.9	25.5	4.2
Child-Pugh classification	A	A	A
Liver stiffness (kPa)	N.D.	32	8.4
HCV RNA (logIU/mL)	7.1	4.8	4.6
HCV genotype	1b	1b	1b
NS5A RASs at L31/Y93	Y93H 33%	Y93H>=99%	None
IL28B rs8099917	TT	unknown	unknown

RASs, resistance-associated substitutions.

## CASE 2

A 49-year-old man was diagnosed with HCV GT1b infection 10 years ago. He has mild constitutional jaundice with a history of brain contusion from a traffic accident and received a blood transfusion at age 17. He denied other risk factors for HCV infection, including tattoos or intravenous drug use. He is a social drinker with no family history of HCV infection. He had a medical history of right upper limb tremor and used clonazepam (0.5 mg daily) to treat it. He also took ursodeoxycholic acid (600 mg daily), spironolactone (25 mg daily) and shakuyakukanzoto extract granules (2.5 g daily).

He was an interferon-treatment naïve patient. At the previous hospital, he was treated with ledipasvir (90 mg daily) and sofosbuvir (400 mg daily) in 2016. However, this treatment was discontinued at 14 days because of hyperCPKemia (2194 IU/L). HCV RNA disappeared at week 4 but relapsed at week 12 after stopping the treatment. He was retreated with grazoprevir (100 mg daily) and elbasvir (50 mg daily) at 13 months after the initial DAA treatment. The laboratory data at the start of retreatment are shown in Table 1. Although a liver biopsy was not performed, his liver stiffness and low platelet counts indicated cirrhosis. HCV NS5A-L31 and -Y93 sequencing using a real-time PCR system and a cycling probe assay [17] revealed that he had Y93H at > 99% as a HCV NS5A RAS. He received full doses of both grazoprevir and elbasvir for 12 weeks, and no adverse events were observed. The eGFR did not change during the therapy. RVR and SVR12 were achieved (Figure 2B).

## CASE 3

A 76-year-old man was referred to our hospital that was diagnosed with HCV GT1b infection and nephrotic syndrome 11 years ago. He did not have surgery, blood transfusions, tattoos, or abuse of drugs at that time. He did not drink alcohol and had no family history of HCV infection. He had a medical history of drug-induced liver injury of benzbromarone [18–20] and received a transfusion for renal anemia 9 years ago.

He received dialysis shunt surgery on the left forearm in 2015. He was an interferon-treatment naïve patient. He was treated with HCV NS5A inhibitor ombitasvir (25 mg)/HCV NS3/4A inhibitor paritaprevir (150 mg)/ritonavir (100 mg) daily in 2016 [21]. However, this treatment was discontinued on day 12 because of renal dysfunction (serum creatinine 8.23 mg/dL) with hyperpotassemia (serum potassium 5.6 mEq/L). HCV RNA disappeared at week 4 but relapsed on week 8 after stopping the treatment. Renal dialysis was performed three times weekly one month later after stopping the treatment (Figure 2C).

The laboratory data at the start of retreatment are shown in Table 1. He had no sign of cirrhosis. HCV NS5A-L31 and -Y93 sequencing using a real-time PCR system and a cycling probe assay [17] revealed he had no HCV NS5A RASs. He began retreatment with grazoprevir (100 mg daily) and elbasvir (50 mg daily) at 13 months after the initial DAA treatment. He also took ursodeoxycholic acid (600 mg daily), rabeprazole sodium (10 mg daily), carvedilol (10 mg daily), losartan potassium (2.5 mg daily)/hydrochlorothiazide (12.5 mg daily) and febuxostat (10 g daily). He received full doses of both grazoprevir and elbasvir for 12 weeks, and no adverse events were observed. RVR and SVR12 were achieved (Figure 2C).

## DISCUSSION

In the present report, we demonstrated three “difficult-to-treat” GT1b patients who previously discontinued initial HCV NS5A inhibitor-including regimens, that were successfully treated by a 12-week regimen of combination of grazoprevir and elbasvir. Cases 1, 2 and 3, respectively, had histories of VT, hyperCPKemia and renal dysfunction as adverse events due to the initial HCV NS5A inhibitor-including regimens. Case 2 also had cirrhosis.

Previously, we reported that 12-week combination regimen of ledipasvir and sofosbuvir is an effective option for HCV GT1b patients without treatment-emergent HCV NS5A RASs, who discontinue the combination of daclatasvir and asunaprevir within 4 weeks [16]. In the present study, we showed the 12-week combination regimens of grazoprevir and elbasvir is an effective retreatment option for HCV GT1b patients with or without HCV NS5A RASs, who discontinue the initial interferon-free treatment with HCV NS5A inhibitor-including regimens due to adverse events within 2 weeks. We do not know whether these RASs in cases 1 and 2 were treatment-emergent HCV NS5A RASs because we did not measure HCV RASs in cases 1 and 2 before the initial interferon-free treatment with the combination of ledipasvir and sofosbuvir [6].

In the American Association for the Study of the Liver Diseases (AASLD) and Infectious Diseases Society of America (IDSA) HCV guidelines, a 12-week regimen of a daily fixed-dose combination of elbasvir (50 mg)/grazoprevir (100 mg) is one of the recommended regimens in the treatment-naïve HCV GT1b patients with or without compensated cirrhosis [22]. In this guideline [22], a 12-week regimen of daily fixed-dose combination of sofosbuvir (400 mg)/velpatasvir (100 mg)/NS3 inhibitor voxilaprevir (100 mg) is one of the recommended retreatment-regimens in NS5A inhibitor DAA-experienced GT1 patients with or without compensated cirrhosis. For the retreatment of sofosbuvir-containing regimen-experienced, GT1b patients with or without compensated cirrhosis, a 12-week regimen of a daily fixed-dose combination of NS3 inhibitor glecaprevir (300 mg)/NS5A inhibitor pibrentasvir (120 mg) or a 12-week regimen of a daily fixed-dose combination of sofosbuvir (400 mg)/velpatasvir (100 mg) is recommended [22]. The guidelines from the Asian Pacific Association for the Study of the Liver (APASL) also recommend that RASs with HCV NS5A regions should be examined before starting any retreatments in patients who failed to response to the HCV NS5A inhibitor-including regimens [2].

In the present report, all cases experienced a short-duration (within 2 weeks) of the initial interferon-free treatment with HCV NS5A inhibitor-including regimens. It also demonstrated that the 12-week retreatment with grazoprevir and elbasvir may be useful as an alternative treatment option. In combination regimen of grazoprevir and elbasvir plus ribavirin for patients with chronic hepatitis C virus GT1 infection after failure of peginterferon and ribavirin with an earlier-generation NS3/4A inhibitor, SVR rates were 96.2% (76/79) [23]. In Japan, other combinations such as glecaprevir/pibrentasvir or sofosbuvir/velpatasvir/ribavirin will be available and will be effective in 2018 for retreatment of such patients. Although these regimens will be first treatment option for retreatment of such patients, the role of the different options will occasionally need to be balanced based on cost, adverse events and drug availability in the different regions where these regimens become available [24].

In our institute, 16 patients started to be treated by the combination regimen of grazoprevir and elbasvir by 31 March 2017. Of these 16 patients, only three patients who has been presented in the present study had experienced the previous failure of interferon-free therapy. All these patients achieved SVR. At present, we do not know whether the retreatment with grazoprevir and elbasvir is also effective for patients who discontinue HCV NS5A inhibitor-including regimens after 2 weeks of treatment. Although the number of patients who discontinue HCV NS5A inhibitor-including regimens within 2 weeks seems to be small, our study could provide some important information for clinicians to manage chronic hepatitis C patients who discontinued the HCV NS5A inhibitor-including regimens due to adverse events within short duration.

In the sofosbuvir-including regimen, VT is a complication, and combination of ledipasvir and sofosbuvir is associated with an increased risk of heart-related events [25]. In our report, patient that have had events of VT during the combination of ledipasvir and sofosbuvir, were successfully retreated with the combination of grazoprevir and elbasvir. We also demonstrated that the combination of grazoprevir and elbasvir can be used safely in patients under haemodialysis.

This retreatment regimen had no serious adverse events in these cases. The treatment response was excellent despite the existence of treatment-emergent HCV NS5A RASs but may be related to the short duration of initial interferon-free treatment. In our previous report [15], one patient who discontinued the combination of daclatasvir and asunaprevir due to viral breakthrough at week 10 and had a treatment-emergent HCV NS5A RAS-Y93H did not achieve SVR with the combination retreatment of sofosbuvir and ledipasvir for 12 weeks. The present study supported retreatment with the combination of grazoprevir and elbasvir, but it should be avoided by HCV GT1b-infected patients who discontinued the initial interferon-free treatment with HCV NS5A inhibitor-including regimens at more than 4 weeks after the commencement of the initial treatment [16].

## CONCLUSIONS

Retreatment with grazoprevir and elbasvir successfully eradicated HCV RNA in three patients without any serious adverse events. This combination might be an effective retreatment option for HCV GT1b patients who discontinue the initial interferon-free treatment HCV NS5A inhibitor-including regimens due to adverse events within 2 weeks.
